# Enhancing Unifocalization With Saline Inflation Testing and a Handmade Valved Conduit in Major Aortopulmonary Collateral Arteries

**DOI:** 10.1016/j.atssr.2025.04.019

**Published:** 2025-05-16

**Authors:** Akio Ikai, Keiichi Hirose, Mizuhiko Ishigaki, Sung-Hae Kim, Kisaburo Sakamoto, Hiroki Ito

**Affiliations:** 1 Pulmonary Hemodynamics Research Division, Department of Clinical Research, Research Support Center, Shizuoka General Hospital, Shizuoka, Japan; 2 Department of Cardiovascular Surgery, Mt. Fuji Shizuoka Children’s Hospital, Shizuoka, Japan; 3 Department of Cardiology, Mt. Fuji Shizuoka Children’s Hospital, Shizuoka, Japan

## Abstract

Unifocalization of pulmonary atresia with ventricular septal defect and major aortopulmonary collateral arteries is technically challenging because of the differing embryological development of the central pulmonary artery, major aortopulmonary collateral arteries, and parenchymal pulmonary arteries. We performed unifocalization using a saline “inflation test” not only to prevent kinking of the vessels, but also to identify differences in extensibility to intergrade uniform vessels. Moreover, right ventricular outflow tract reconstruction using a handmade valved conduit was performed to facilitate easy access to the catheter intervention to maintain a uniform vascular network. This approach offers a potential solution for the treatment of complex conditions.

Treating pulmonary atresia with ventricular septal defect (PAVSD) with major aortopulmonary collateral arteries (MAPCAs) is challenging because of the involvement of the central pulmonary artery (PA), MAPCAs, and parenchymal PAs, each of which develops through distinct embryological processes.^1^ Parenchymal PAs, which are influenced by the pulmonary mesenchyme, are more distensible. This report describes unifocalization, focusing on vessel extensibility based on intraoperative saline inflation tests.

## Technique

**Figure 1:**
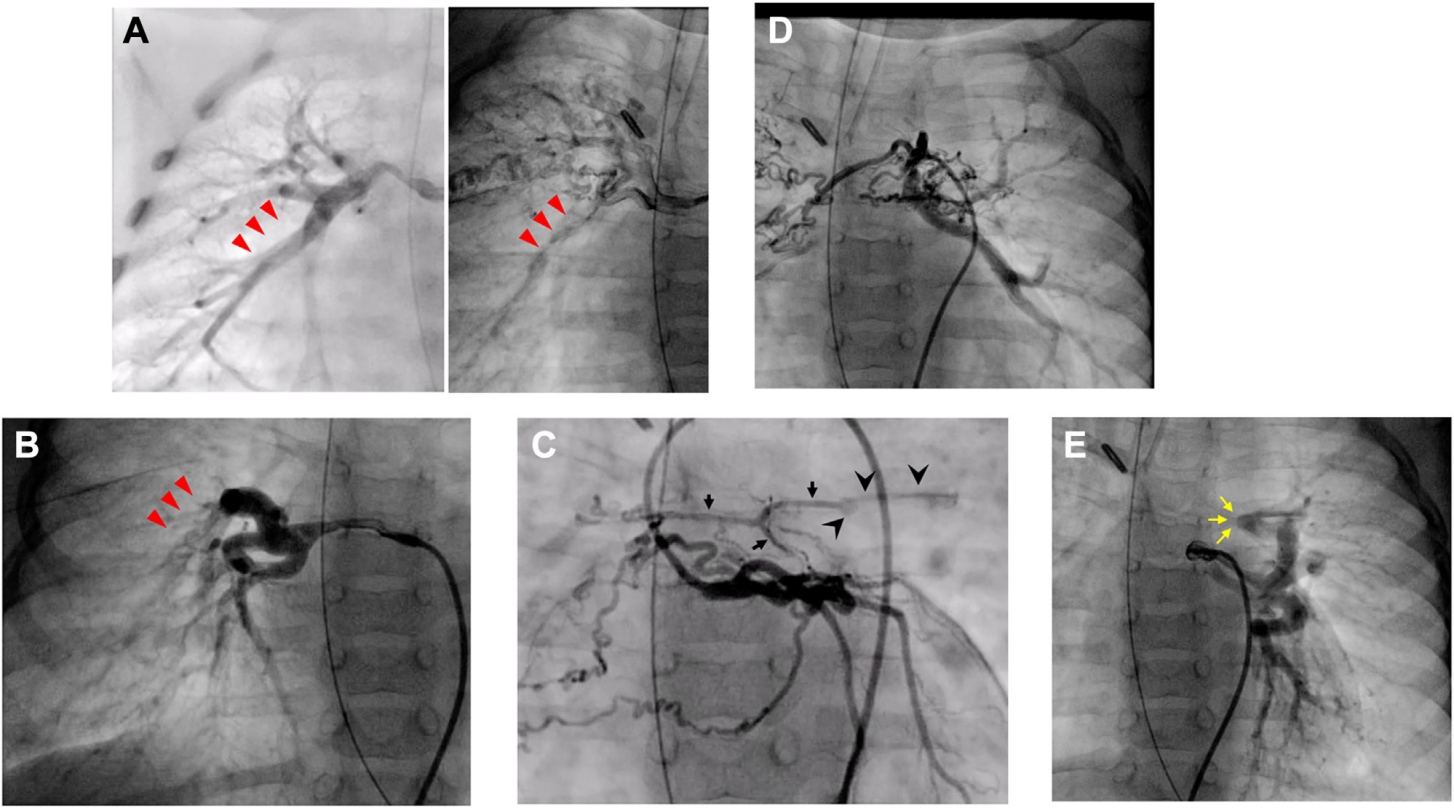
Selective angiography. (A) Right upper major aortopulmonary collateral artery (MAPCA) (red arrowheads) before and after shunt. (B) Right lower MAPCA. Red arrowheads indicate the same vessel shown in (A). (C) Coronary angiography showing a diminutive central pulmonary artery (PA) (black arrows) and a left parenchymal pulmonary artery (black arrowheads). (D) Left upper MAPCA. (E) Lower left MAPCA showing the hilum portion formed by the left parenchymal pulmonary artery (yellow arrows).

This study was approved by the institutional review board of Shizuoka General Hospital (SGHIRB#2024008) on May 17, 2024. Patient consent was obtained. A 1.6-year-old boy weighing 8.4 kg with PAVSD and MAPCAs underwent surgery. He had 4 MAPCAs (2 on each side) originating from the descending aorta. Prior surgery at another institution involved a right-sided modified Blalock-Taussig shunt. Both right MAPCAs ran behind the bronchus. The left lower MAPCA had an inadequate dual supply that formed the left pulmonary hilum, and its connection with the diminutive central PA was visible on coronary angiography (Figure 1) (Supplemental Video 1).

**Figure 2:**
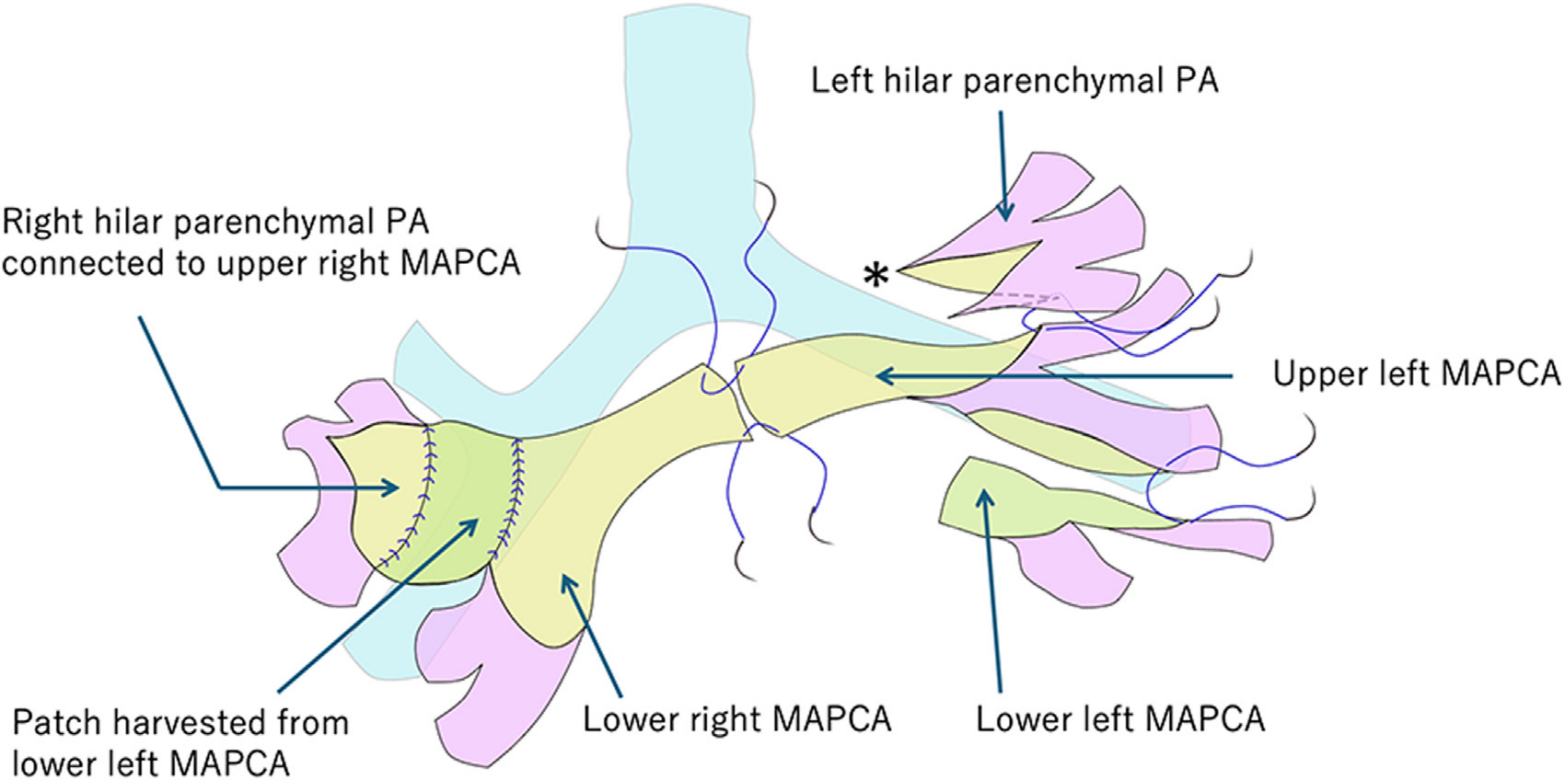
Schematic view of unifocalization. In the right hilum, a patch harvested from the left upper major aortopulmonary collateral artery (MAPCA) was interposed to create a central pulmonary artery (PA) platform.

A handmade tricuspid-valved expanded polytetrafluoroethylene conduit (16 mm diameter) was created for complete repair, and single-stage unifocalization was performed using the Stanford technique (Supplemental Video 2).^2^ The saline “inflation test” identified a more distensible vessel uniformly beyond the stenosis, and anastomoses for these vessels were performed using 8-0 polypropylene sutures. The nonextensible narrow central PA was resected, and the platform of the central PA was reconstructed using left and right MAPCAs (Figure 2).

The intraoperative flow study demonstrated a mean PA pressure of 31 mm Hg at 3 L/min/m^2^, prompting a palliative right ventricular outflow tract reconstruction with a prefabricated valve of 16 mm and a ringed 10-mm composite conduit using the “dunk” technique. Vascular clips controlled the graft size to maintain a mean PA pressure of 25-30 mm Hg to avoid unexpected pulmonary vascular effects due to excessive blood flow.

The postoperative course ran as follows. Planned balloon angioplasty was performed 6 months later, followed by unclipping of the conduit. At age 3.5 years and weighing 11 kg, the patient underwent ventricular septal defect (VSD) closure and conduit replacement. The postoperative right ventricular and aortic pressures were 44/1 mm Hg and 80/51 mm Hg, respectively. (Supplemental Video 3).

## Comment

In the unifocalization procedure, an “inflation test” was performed to identify the site of stenosis in MAPCA after dissection. We have extended the concept of the inflation test not only to identify stenosis sites but also to check the extensibility of the vessels in order to build up a uniform vascular bed. The aim of our unifocalization procedure was to establish the continuity of the PA with this uniform extensibility. In this concept of unifocalization, the presence or absence of a central PA does not matter, as long as there is a normal distensible vessel that can be formed at the pulmonary hilum.^2^^,^^3^

However, this anastomosis is technically challenging and may lead to reintervention. Indeed, in the Stanford group, more than half of the patients indicated the need for reintervention.^4^^,^^5^ Interestingly, the Stanford group demonstrated the efficacy of balloon PA angioplasty for stenotic lesions after complete repair.^6^ There is a risk of pseudoaneurysm with palliative right ventricular outflow tract reconstruction in favor of catheter intervention,^3^^,^^7^ which we avoided with the dunk method. In our case, the handmade expanded polytetrafluoroethylene valve conduit proved to be effective, as it was able to regulate blood flow enough to withstand balloon angioplasty. Since 2017, 30 patients with PAVSD and MAPCA have been treated using this concept of unifocalization at our institution. Twenty-six patients, excluding 3 early death cases, achieved complete repair. Follow-up catheterization was performed in 24 of the 26 patients who underwent VSD closure. In the primary group, all patients were evaluated postoperatively. In the staged group, all patients received catheter evaluation after initial unifocalization, and 7 of the 9 who eventually underwent VSD closure had additional assessment after that procedure. The median right ventricular pressure was 43 mm Hg, and the right ventricular-to-aortic pressure ratio was 0.57, indicating favorable hemodynamics. Balloon angioplasty for anastomotic stenosis was required in 45.6% of patients within 1 year of unifocalization, but no surgical reintervention was needed after VSD closure. These findings suggest that structured catheter surveillance enables timely intervention and helps sustain pulmonary artery patency. This concept of unifocalization may lead more patients to the VSD closure.
